# Unmet needs and future perspectives in hydroxychloroquine retinopathy

**DOI:** 10.3389/fmed.2023.1196815

**Published:** 2023-06-09

**Authors:** Imran H. Yusuf, Peter Charbel Issa, Seong Joon Ahn

**Affiliations:** ^1^Oxford Eye Hospital and Nuffield Department of Clinical Neurosciences, University of Oxford, John Radcliffe Hospital, Oxford, United Kingdom; ^2^Department of Ophthalmology, Hanyang University Hospital, Hanyang University College of Medicine, Seoul, Republic of Korea

**Keywords:** hydroxychloroquine, perspectives, retinal toxicity, screening, unmet needs, natural history, definition

## Abstract

Retinopathy is a well-recognized toxic effect of hydroxychloroquine treatment. As hydroxychloroquine retinopathy is potentially a vision-threatening condition, early detection is imperative to minimize vision loss due to drug toxicity. However, early detection of hydroxychloroquine retinopathy is still challenging even with modern retinal imaging techniques. No treatment has been established for this condition, except for drug cessation to minimize further damage. In this perspective article, we aimed to summarize the knowledge gaps and unmet needs in current clinical practice and research in hydroxychloroquine retinopathy. The information presented in this article may help guide the future directions of screening practices and research in hydroxychloroquine retinopathy.

## 1. Introduction

Hydroxychloroquine, a widely used drug for the treatment of numerous rheumatologic and dermatologic disorders (e.g., rheumatoid arthritis and systemic lupus erythematosus), may cause a form of retinal toxicity called hydroxychloroquine retinopathy. The pathogenic mechanism of retinal toxicity is poorly understood. Impaired autophagy and defective phagocytosis of photoreceptor outer segments has been suggested as the pathogenic mechanism of chloroquine/hydroxychloroquine toxicity (1, 2), whereas the role of melanin, whether harmful or protective, remains controversial. Hydroxychloroquine retinopathy is reported to be irreversible, progressive, and vision threatening if detected late. Substantial progress has been made in the diagnosis of hydroxychloroquine retinopathy with modern retinal imaging techniques such as spectral-domain optical coherence tomography (OCT) and fundus autofluorescence (FAF) imaging. National guidelines also play a crucial role in retinopathy detection by identifying high-risk patients and providing recommendations on screening modalities and frequency. The guidelines recommend four screening tests: OCT, FAF, automated visual fields, and multifocal electroretinogram (mfERG). The most recent AAO guidelines designated OCT and automated visual fields as primary screening tests (3).

Despite efforts to standardize screening practices and improved knowledge of disease phenotypes and natural disease course, early detection and management of hydroxychloroquine retinopathy remain challenging. This perspective article aimed to highlight the unmet needs in hydroxychloroquine retinopathy, including the consensus definition of retinal toxicity, hydroxychloroquine blood levels and pharmacogenomics, animal models of disease, roles of ophthalmologists, and use of artificial intelligence (AI) in screening.

## 2. Toward a consensus definition for retinal toxicity

Published studies and clinical guidelines provide substantially different definitions for retinal toxicity and discrepant data on the sensitivity of screening tests. In particular, previous studies have shown disparities in the role of visual field testing (4, 5), leading to divergent recommendations. Elucidating the early natural history of the disease and its manifestations in mainstream diagnostic tests are central in reaching a consensus on the definition of retinal toxicity.

The most recent American Academy of Ophthalmology (AAO) guidelines (2016) specify a broad diagnostic criterion for toxicity: “at least one objective test abnormality confirming a subjective test abnormality.” However, the most recent Royal College of Ophthalmologists criteria require two abnormal test results to identify “definite toxicity,” which must include at least one objective structural test result but need not include visual field testing if both OCT and FAF imaging provide objective evidence of toxicity. This disparity is based on the role and cost of visual field testing, with recent data suggesting that automated visual field testing may fail to detect scotomas despite structural changes on OCT (4). In contrast, some reported cases showed characteristic ring scotoma on visual field testing with no or subtle changes on OCT (5). As FAF imaging may not detect very early disease, visual field testing remains an important primary test in the 2016 AAO guideline (3, 6).

Because OCT is highly sensitive in detecting characteristic outer retinal changes in the parafoveal or pericentral areas, it has a central role in defining toxicity. According to recent evidence, retinal toxicity can be recognized using OCT alone, through the identification of outer retinal thinning on several OCT systems, as well as typical photoreceptor or retinal pigment epithelial damage on B-scans. Given the rapid image acquisition of OCT without the need for pupil dilation, its acceptability to patients, and its relatively low cost, future definitions of toxicity will likely be mainly based on objective structural data from OCT images. Consequently, establishing an OCT-based consensus definition for toxicity may be an important short-term goal.

**Figure 1:**
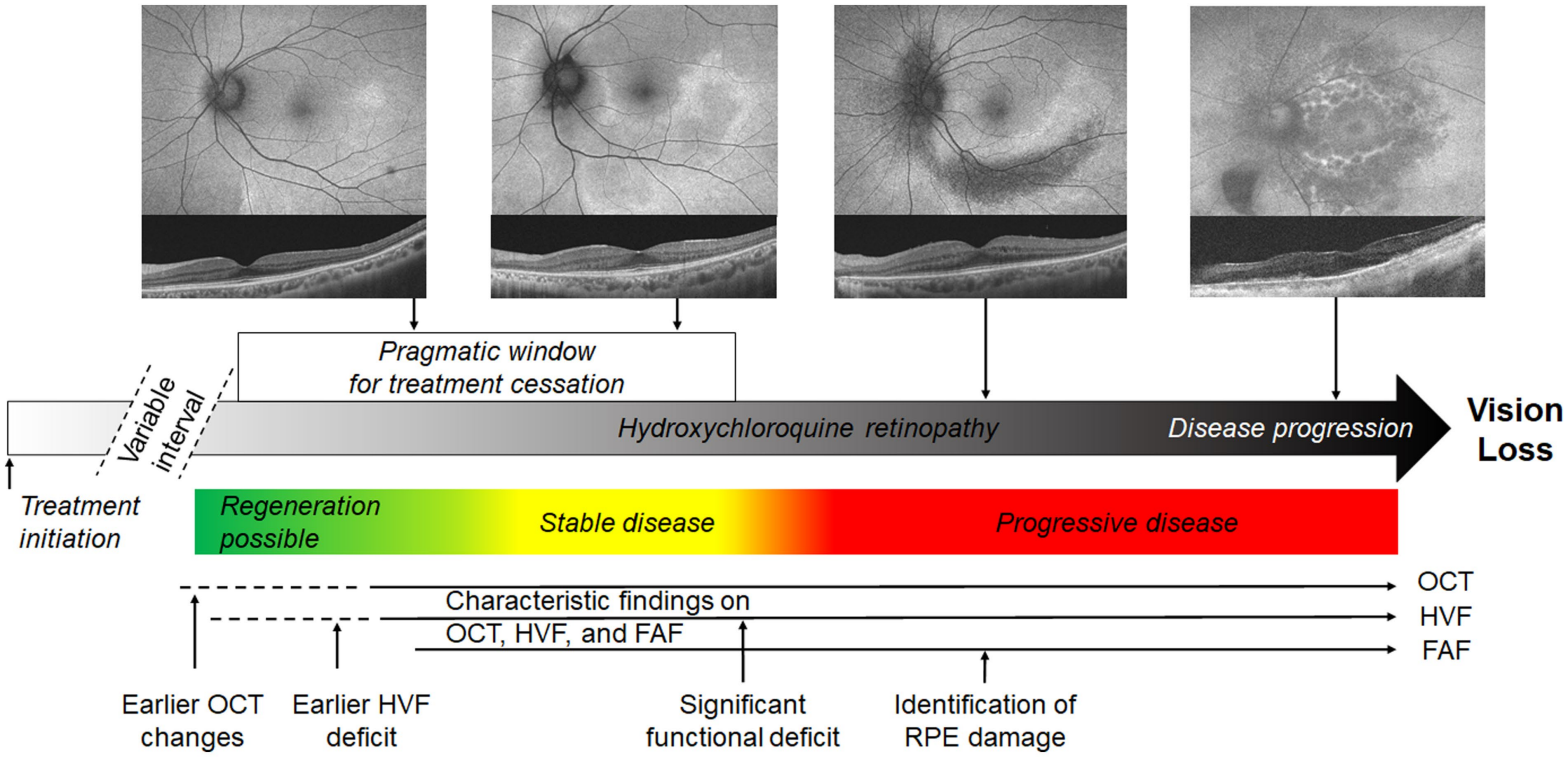
Schematic representation of possible definitions of toxicity in hydroxychloroquine retinopathy. The pragmatic interval of treatment cessation is indicated by the white box, defined as the interval between sufficient evidence of retinopathy to the point at which a significant functional deficit occurs. This limit is not specified and requires a consensus. Colored boxes represent the most likely disease behavior after treatment cessation, although regeneration is not invariable in early disease. The approximate intervals at which disease is detectable using mainstream screening tests is indicated. FAF, fundus autofluorescence; OCT, optical coherence tomography; HVF, automated visual fields.

The definition of toxicity may be formed on the basis of a few critical points in the disease course (Figure 1): (i) the threshold at which structural (e.g., OCT) abnormalities are detected; (ii) the threshold at which functional deficits are detectable. A consensus on the definition of retinopathy would enable standardized testing protocols, diagnostic criteria and comparisons between study populations. Further, the threshold for management should be carefully defined for patients with hydroxychloroquine retinopathy. Clinicians may allow the continuation of hydroxychloroquine for the primary treatment indication until the agreed threshold is reached, even if retinal toxicity is present on the basis of retinal imaging findings alone. In clinical practice, the drug may be discontinued at a threshold at which disease progression does not occur, provided that functional deficits are early or mild and do not affect daily activities such as driving and reading.

Further discussion is required to reach a consensus on the terms used to describe hydroxychloroquine retinopathy, particularly in early disease. A clearer description of the early natural history of disease, in particular relating to distribution and progression in the context of mainstream diagnostic tests, may help to further refine the classification system.

A few studies have noted discrepancies in retinopathy severity when using different imaging modalities (7). Currently, the degree of photoreceptor damage around the fovea is used for distinguishing between early and moderate stages; however, the appropriate test for classification and whether the degree of damage precisely corresponds to the extent of retinopathy are unclear. The staging of disease severity also depends on the sensitivity of diagnostic tests. Currently, no single test can be universally applied for the precise classification of retinopathy. For example, subtle outer retinal changes may not be identifiable using FAF imaging (8), and distinguishing between early and moderate stages solely based on OCT B-scans may be challenging. Alternatively, the area or extent of outer retinal damage can be used to classify the severity of retinopathy. A consensus should be reached on disease classification based on commonly used modalities to standardize the nomenclature among studies and further characterize the disease, thereby allowing a more direct comparison of study results.

## 3. Hydroxychloroquine levels in blood

Serum hydroxychloroquine levels have been evaluated with respect to adverse drug effects. A previous study measured blood hydroxychloroquine levels after a loading phase with variable doses in three groups of patients with rheumatoid arthritis (9). The results revealed a correlation between serum hydroxychloroquine levels and gastrointestinal adverse events (9).

Although measurement of blood hydroxychloroquine levels is relevant in determining clinical efficacy, its role in ascertaining the chronic toxic effects of drugs is unclear. A single high measurement of serum hydroxychloroquine level may not necessarily reflect steady-state pharmacokinetics; therefore, a series of measurements prospectively performed over many years is required to evaluate the utility of blood hydroxychloroquine levels as a risk factor for hydroxychloroquine retinopathy. However, in patients undergoing extremely high-dose hydroxychloroquine adjuvant chemotherapy over shorter durations, often as part of clinical trials (10–15), measurement of blood hydroxychloroquine levels may be valuable in the short term for evaluating whether a particular measurement may predict hydroxychloroquine retinopathy development.

Two recent studies on the relationship between serum hydroxychloroquine level and retinopathy development reported conflicting results (16, 17). In a case–control study involving 23 patients with confirmed hydroxychloroquine retinopathy and 547 controls, blood hydroxychloroquine levels were not significant predictors in univariate analysis (16). However, in another study that identified 23 (of 537) patients with confirmed retinopathy, serum hydroxychloroquine levels (mean or maximum) predicted later retinopathy (17). Further data analysis revealed that patients in the lowest tertile of time-adjusted serum hydroxychloroquine levels (0–739 ng/mL) had a 1.2% risk of retinopathy, those in the middle tertile (740–1,180 ng/mL) had a 3.1% risk, and those in the highest tertile (1,181–3,466 ng/mL) had an 8.5% risk, and the differences were statistically significant for the trend. Logistic regression analysis identified that the relationship between serum hydroxychloroquine levels and retinopathy remained significant after adjustment for therapy duration (18). Although the level was reported to be useful for prediction of retinopathy development, its clinical utility for prediction of future progression after drug cessation has not been validated. As the retinopathy is known to progress in advanced stages even after drug cessation, its predictive role in eyes with advanced disease stages may be limited and should be investigated further in future studies.

There have been a few reports on the rapid-onset retinal toxicity of hydroxychloroquine, in which cases occurred within 3 years of use (19, 20). Ozawa et al. reported abnormally high blood levels of hydroxychloroquine in one patient (20). The result suggests that hydroxychloroquine blood level may be useful for understanding and predicting rapid-onset disease.

Considering the established risk factors for retinal toxicity, further studies are required to determine the significance of serum hydroxychloroquine levels in definition or prediction of the risk of retinopathy. Furthermore, the relationship between the daily dose and blood levels of hydroxychloroquine should be validated. If this relationship is proven, serum hydroxychloroquine levels may be used to adjust the daily doses to achieve a balance between efficacy in treating the primary disease and the risk of retinal toxicity. This may help individualize hydroxychloroquine treatment to maximize the therapeutic effects while minimizing the toxicity risk.

## 4. Pharmacogenomics

Patients with identical exposure to hydroxychloroquine may have differing susceptibility to retinopathy. Some patients may develop retinopathy even with low-dose hydroxychloroquine use (5) or at a much faster rate than anticipated (21). This disparity in susceptibility may be explained by disease modifiers, including genetic or environmental factors. Pharmacogenomics investigates how the genetic factors affects a person’s response to drugs and even drug-related side effects. The identification of risk alleles for retinal toxicity may be useful in further reducing the risk of toxicity and the cost of screening by seeking alternative medications for high-risk patients.

An initial report suggested that certain *ABCA4* missense variants associated with Stargardt disease may predispose patients to hydroxychloroquine-or chloroquine-induced retinal toxicity (22), although a further study demonstrated a protective effect (23). In another study, 99 patients with >5 years of hydroxychloroquine exposure underwent genetic testing of 960,919 single nucleotide polymorphisms, and 13 common macular dystrophy genes were sequenced in a separate cohort of 44 cases and 53 controls (24). Furthermore, whole-exome sequencing was performed in 16 cases and 17 controls for all genes associated with retinal dystrophy, chloroquine pathway metabolism, and autophagy. In this large series, genetic tests did not reveal an association with hydroxychloroquine retinopathy.

Large collaborative studies using an unbiased approach, such as a genome-wide association study, may be required to detect genetic traits that confer an increased risk. The success of such studies depends on the allele frequency of unknown genetic variants that influence toxicity, the strength of their effect, and the sample size. Even large collaborative studies may fail to identify a genetic locus if the effect size is small. Studies will likely require age-matched controls treated with hydroxychloroquine but without toxicity as confirmed by rigorous screening procedures, as well as age-matched controls without hydroxychloroquine exposure.

If a genetic locus that confers risk of hydroxychloroquine-induced retinal toxicity is identified, the relative influence of this predictive genetic locus should be evaluated with respect to other known risk factors for retinopathy (e.g., drug use duration and tamoxifen use). A further promise of pharmacogenomic investigation is improved understanding of the pathophysiology of hydroxychloroquine retinopathy. Identification of candidate loci is likely to motivate a variety of functional studies to further delineate the influence of such loci on drug pharmacokinetics or on the local effect of hydroxychloroquine on retinal pigment epithelial or photoreceptor cells.

## 5. Development of animal or cellular models

The development of specific therapies for hydroxychloroquine retinopathy, to prevent disease formation or protect against further degeneration, has been hampered by the lack of validated disease models. Rodents lack an anatomical macula, which is the classic site of retinopathy. An experiment with albino rats identified a dose-dependent decrease in B-wave amplitude on full-field electroretinography after chloroquine exposure (25). In the 1970s, rhesus monkeys were intramuscularly injected with chloroquine for 4.5 years without causing fundus, retinal angiographic, or electrophysiological abnormalities. The use of OCT and FAF imaging may enable a more sensitive and earlier detection of the disease, considerably shortening the observation period of such studies (26). However, primate studies have not been repeated, perhaps because the perceived rarity of retinopathy does not justify the resources required for these investigations. The existence of validated disease models would enable investigation into the role of potential therapies in stopping or slowing down disease progression. The increasing use of hydroxychloroquine, emerging prevalence data, and ability to detect retinopathy at earlier stages may together lead to renewed interest in establishing disease models to better characterize the pathogenesis of hydroxychloroquine retinopathy and to develop new therapies.

*In vitro* or cell-based models (ARPE19) may be useful for determining the effect of hydroxychloroquine exposure on cellular omics (gene expression) in the short term (27). These models may help elucidate the pathways to toxicity. Single-cell expression assays (e.g., RNA-Seq) can enhance the understanding of cellular responses to drug exposure. However, cell culture techniques cannot be used to model clinically relevant chronic exposure to hydroxychloroquine, or the interdepedence of the retina and RPE which may be relevant in this disease.

## 6. Changing roles of ophthalmologists

The role of ophthalmologists in the management of hydroxychloroquine retinopathy have not been addressed extensively in the literature. Understanding the role of ophthalmologists in hydroxychloroquine retinopathy requires considering the unusual nature of the disease: (i) hydroxychloroquine retinopathy generally occurs many years after therapy initiation (sometimes after >20 years), (ii) ophthalmologists do not prescribe hydroxychloroquine, and (iii) ophthalmologists are not involved in using hydroxychloroquine for treating the primary disease. This scenario helps explain the responsibilities of health-care professionals involved from therapy initiation to drug cessation and those of ophthalmologists, including establishing links with relevant physicians and departments, establishing screening services, training colleagues in data interpretation, improving patient education, and auditing service outcomes.

As hydroxychloroquine can be initiated for various clinical indications, it may be prescribed by several different specialists. Ophthalmologists should provide further information to patients about hydroxychloroquine retinopathy at their first involvement with screening services, including the nature and timing of screening tests.

Ophthalmologists can ensure safe dosing according to the patient’s body weight to reduce the risk of toxicity, and this may require providing recommendations to the prescribing physician. Recommendations on potentially revising the hydroxychloroquine dose after any substantial weight loss may also be helpful. Ophthalmologists will determine the timing of annual screening visits for the evaluation of dosing, renal function, and concurrent tamoxifen use, and this should be communicated to prescribing physicians along with the baseline ophthalmological findings.

The results of screening tests should be communicated to the patient and prescribing physician. If definite retinopathy exists, a recommendation to stop treatment can be made to the prescribing physician, who can subsequently discuss treatment options with the patient. To facilitate this discussion, a description of disease severity (early, moderate, or severe) is helpful. Patients with early retinopathy but with severe systemic disease may elect to continue hydroxychloroquine because the benefits of systemic treatment are immediate (and perhaps more profound) and the toxic effects on the retina are slow. Patients should be actively involved in the decision to stop hydroxychloroquine therapy as guided by clear information from the ophthalmologist and prescribing physician. Considering the functional effects of retinopathy (e.g., on the ability to drive or work) may be helpful. Clear communication between ophthalmologists and patients may also minimize anxiety in at-risk patients.

The ophthalmologist’s role extends to understanding the organizations and individuals responsible for referrals and referral pathways including rheumatologists, dermatologists, and other specialist services. Rheumatologists, dermatologists should be able to access screening services, track the use of screening services in patients at risk under their care, and have access to screening outcomes. Ophthalmologists are responsible for ensuring rigorous screening procedures, including image quality, controlled reporting, and auditing outcomes - as have been established screening for diabetic retinopathy.

On the diagnosis of hydroxychloroquine retinopathy, the ophthalmologist should provide the necessary support depending on each patient’s retinopathy stage, social circumstances, visual function, emotional distress, and ocular and systemic comorbidities. Low-vision services may be required for patients with substantial visual impairment, and registration of visual impairment may be necessary for those with advanced visual field loss. Although further visits would not change the clinical course after drug cessation, they may be necessary for patients who are particularly concerned about disease progression and those with moderate-to-advanced disease. Patients at risk of retinopathy should not be discharged if they fail to attend an appointment, and a fail-safe mechanism is required to ensure that at-risk patients are screened.

## 7. Role of AI in retinopathy screening

Artificial intelligence (AI) analysis of digital retinal images is a rapid and noninvasive method of identifying and characterizing the pathological features of macular and retinal diseases (28). In particular, deep learning algorithm using convolutional neural networks can be developed to extract generalized features from digital images. By using training datasets, these tools enable the recognition of pathology through supervised and unsupervised methods. However, unsupervised techniques may yield novel subclinical imaging biomarkers of disease because the methods are not biased by assumptions (29). The use of AI is particularly suited to screening in which early disease manifestations may be subtle and easily missed by human observers. Convolutional neural networks have been trained to perform comparably to human graders of diabetic retinopathy images (30). OCT interpretation of images has been demonstrated in macular disorders such as age-related macular degeneration and diabetic macular edema (28).

Although only one study has evaluated the utility of AI in hydroxychloroquine retinopathy detection, it has great potential to increase the sensitivity of early retinopathy detection (31). The earliest known OCT finding in hydroxychloroquine retinopathy was localized outer nuclear layer thinning before the development of qualitative changes in the outer retinal layers, more easily detectable by human observers. The subjectivity of OCT image interpretation may explain the conflicting data on the natural history of hydroxychloroquine retinopathy, with some studies indicating that visual field changes may precede OCT abnormalities (5) and others reporting the opposite finding (4). Recent clinical studies have shown that OCT maps of retinal thickness may help human observers detect localized retinal thinning in patients relative to age-matched controls (32). Automated OCT segmentation tools and software may facilitate this process. However, AI may detect the disease at an earlier stage using as yet unknown retinal/OCT biomarkers or through integrated observations of large populations. Accordingly, AI may play a crucial role in further delineating the natural history of early hydroxychloroquine retinopathy based on objective structural outcomes.

AI may facilitate the identification of objective thresholds before which retinopathy is not progressing, permitting patients to continue benefiting from hydroxychloroquine therapy until this threshold is reached. This represents a clinically meaningful, evidence-based, patient-centered endpoint. The use of AI will also minimize inappropriate treatment cessation, which is one of the main risks of screening. Furthermore, patients with very early retinopathy may continue to benefit from hydroxychloroquine therapy if their retinal function is not yet threatened. Moreover, if AI can be harnessed to detect the earliest stage of disease, reclassification of retinopathy stages (normal, preclinical retinopathy [beyond human detection], preperimetric retinopathy [structural deficit but no functional deficit], and retinopathy with functional deficit) would be necessary. Further, the cost of hydroxychloroquine retinopathy screening can be substantially reduced by personalizing the intervals between screening visits, thereby reducing the overall number of screening episodes for a given population of at-risk patients.

The major barrier to the development of AI tools for detecting hydroxychloroquine retinopathy is the number of OCT images required to train an algorithm. Moreover, the clinical manifestations of hydroxychloroquine retinopathy seem to be partially dependent on ethnicity. A previous study with a multiethnic cohort of patients with diabetic retinopathy showed that AI required 100,000 images for training (33). However, for hydroxychloroquine retinopathy, acquiring a dataset of this size would require multinational collaboration and curation.

## 8. Conclusion

No consensus has been reached on the definition and classification of early hydroxychloroquine retinopathy. As early detection of retinopathy remains challenging, personalized screening according to the retinopathy risk based on hydroxychloroquine blood levels or pharmacogenomics could help to further refine screening by identifying patients at greater risk. The roles of ophthalmologists are changing, and better communication with prescribing physicians and patients are important for appropriate management and regular monitoring. Finally, advances in AI and AI-assisted screening programs for retinopathy should be integrated into health-care systems, which require future research.

## Data availability statement

The original contributions presented in the study are included in the article/supplementary material, further inquiries can be directed to the corresponding author.

## Author contributions

IY and SA: conception, design, and data collection. IY, PC, and SA: analysis, interpretation, obtain funding, overall responsibility, and approve the submitted version. All authors contributed to the article and approved the submitted version.

## Funding

This work was supported by National Research Foundation of Korea grants funded by the Korean government (MSIT; NRF-2021R1G1A1013360); the National Institute for Health Research Biomedical Research Centre, Oxford, UK; and the Medical Research Council UK (MR/R000735/1). Knoop Junior Research Fellowship, St. Cross College, University of Oxford.

## Conflict of interest

The authors declare that the research was conducted in the absence of any commercial or financial relationships that could be construed as a potential conflict of interest.

## Publisher’s note

All claims expressed in this article are solely those of the authors and do not necessarily represent those of their affiliated organizations, or those of the publisher, the editors and the reviewers. Any product that may be evaluated in this article, or claim that may be made by its manufacturer, is not guaranteed or endorsed by the publisher.

## Abbreviations

AAO: American Academy of Ophthalmology
AI: artificial intelligence
FAF: fundus autofluorescence
OCT: optical coherence tomography
QoL: quality of life

## References

[1] Mannerström M Mäenpää H Toimela T Salminen L Tähti H. The phagocytosis of rod outer segments is inhibited by selected drugs in retinal pigment epithelial cell cultures. Pharmacol Toxicol. (2001) 88:27–33. doi: 10.1034/j.1600-0773.2001.088001027.x, PMID: 11169158

[2] Yoon YH Cho KS Hwang JJ Lee SJ Choi JA Koh JY. Induction of lysosomal dilatation, arrested autophagy, and cell death by chloroquine in cultured ARPE-19 cells. Invest Ophthalmol Vis Sci. (2010) 51:6030–7. doi: 10.1167/iovs.10-5278, PMID: 20574031

[3] Marmor MF Kellner U Lai TY Melles RB Mieler WF American Academy of Ophthalmology. Recommendations on screening for chloroquine and hydroxychloroquine retinopathy (2016 revision). Ophthalmology. (2016) 123:1386–94. doi: 10.1016/j.ophtha.2016.01.058, PMID: 26992838

[4] Garrity ST Jung JY Zambrowski O Pichi F Su D Arya M. Early hydroxychloroquine retinopathy: optical coherence tomography abnormalities preceding Humphrey visual field defects. Br J Ophthalmol. (2019) 103:1600–4. doi: 10.1136/bjophthalmol-2018-313350, PMID: 30819690

[5] Marmor MF Melles RB. Disparity between visual fields and optical coherence tomography in hydroxychloroquine retinopathy. Ophthalmology. (2014) 121:1257–62. doi: 10.1016/j.ophtha.2013.12.002, PMID: 24439759

[6] Yusuf IH Foot B Lotery AJ. The Royal College of ophthalmologists recommendations on monitoring for hydroxychloroquine and chloroquine users in the United Kingdom (2020 revision): executive summary. Eye (Lond). (2021) 35:1532–7. doi: 10.1038/s41433-020-01380-2, PMID: 33423043 PMC8169737

[7] Ahn SJ Joung J Lee BR. En face optical coherence tomography imaging of the photoreceptor layers in hydroxychloroquine retinopathy. Am J Ophthalmol. (2019) 199:71–81. doi: 10.1016/j.ajo.2018.11.003, PMID: 30448463

[8] Ahn SJ Joung J Lee BR. Evaluation of hydroxychloroquine retinopathy using ultra-Widefield fundus autofluorescence: peripheral findings in the retinopathy. Am J Ophthalmol. (2020) 209:35–44. doi: 10.1016/j.ajo.2019.09.008, PMID: 31526798

[9] Munster T Gibbs JP Shen D Baethge BA Botstein GR Caldwell J. Hydroxychloroquine concentration-response relationships in patients with rheumatoid arthritis. Arthritis Rheumatol. (2002) 46:1460–9. doi: 10.1002/art.10307, PMID: 12115175

[10] Poklepovic A Gewirtz DA. Outcome of early clinical trials of the combination of hydroxychloroquine with chemotherapy in cancer. Autophagy. (2014) 10:1478–80. doi: 10.4161/auto.29428, PMID: 24991829 PMC4203528

[11] Mahalingam D Mita M Sarantopoulos J Wood L Amaravadi RK Davis LE. Combined autophagy and HDAC inhibition: a phase I safety, tolerability, pharmacokinetic, and pharmacodynamic analysis of hydroxychloroquine in combination with the HDAC inhibitor vorinostat in patients with advanced solid tumors. Autophagy. (2014) 10:1403–14. doi: 10.4161/auto.29231, PMID: 24991835 PMC4203517

[12] Rangwala R Chang YC Hu J Algazy KM Evans TL Fecher LA. Combined MTOR and autophagy inhibition: phase I trial of hydroxychloroquine and temsirolimus in patients with advanced solid tumors and melanoma. Autophagy. (2014) 10:1391–402. doi: 10.4161/auto.29119, PMID: 24991838 PMC4203516

[13] Vogl DT Stadtmauer EA Tan KS Heitjan DF Davis LE Pontiggia L. Combined autophagy and proteasome inhibition: a phase 1 trial of hydroxychloroquine and bortezomib in patients with relapsed/refractory myeloma. Autophagy. (2014) 10:1380–90. doi: 10.4161/auto.29264, PMID: 24991834 PMC4203515

[14] Wolpin BM Rubinson DA Wang X Chan JA Cleary JM Enzinger PC. Phase II and pharmacodynamic study of autophagy inhibition using hydroxychloroquine in patients with metastatic pancreatic adenocarcinoma. Oncologist. (2014) 19:637–8. doi: 10.1634/theoncologist.2014-0086, PMID: 24821822 PMC4041680

[15] Xu R Ji Z Xu C Zhu J. The clinical value of using chloroquine or hydroxychloroquine as autophagy inhibitors in the treatment of cancers: a systematic review and meta-analysis. Medicine. (2018) 97:e12912. doi: 10.1097/MD.0000000000012912, PMID: 30431566 PMC6257684

[16] Lenfant T Salah S Leroux G Bousquet E Le Guern V Chasset F. Risk factors for hydroxychloroquine retinopathy in systemic lupus erythematosus: a case-control study with hydroxychloroquine blood-level analysis. Rheumatology (Oxford). (2020) 59:3807–16. doi: 10.1093/rheumatology/keaa157 32442312 PMC8186841

[17] Petri M Elkhalifa M Li J Magder LS Goldman DW. Hydroxychloroquine blood levels predict hydroxychloroquine retinopathy. Arthritis Rheumatol. (2020) 72:448–53. doi: 10.1002/art.41121, PMID: 31532077 PMC7050401

[18] Petri M Elkhalifa M Li J Magder LS Goldman DW. Reply to letter to editor: do hydroxychloroquine blood levels and dosage identify the same or different populations at risk for retinopathy? Arthritis Rheumatol. (2020) 72:2166. doi: 10.1002/art.41462

[19] Marmor MF. COVID-19 and chloroquine/hydroxychloroquine: is there ophthalmological concern? Am J Ophthalmol. (2020) 213:A3–4. doi: 10.1016/j.ajo.2020.03.028, PMID: 32247518 PMC7270810

[20] Ozawa H Ueno S Ohno-Tanaka A Sakai T Hashiguchi M Shimizu M. Ocular findings in Japanese patients with hydroxychloroquine retinopathy developing within 3 years of treatment. Jpn J Ophthalmol. (2021) 65:472–81. doi: 10.1007/s10384-021-00841-9, PMID: 34014447

[21] Stern EM Johnson JS Mazzulla DA. Highly accelerated onset of hydroxychloroquine macular retinopathy. Ochsner J. (2017) 17:280–3. PMID: 29026363 PMC5625990

[22] Shroyer NF Lewis RA Lupski JR. Analysis of the ABCR (ABCA4) gene in 4-aminoquinoline retinopathy: is retinal toxicity by chloroquine and hydroxychloroquine related to Stargardt disease? Am J Ophthalmol. (2001) 131:761–6. doi: 10.1016/S0002-9394(01)00838-8, PMID: 11384574

[23] Grassmann F Bergholz R Mandl J Jagle H Ruether K Weber BH. Common synonymous variants in ABCA4 are protective for chloroquine induced maculopathy (toxic maculopathy). BMC Ophthalmol. (2015) 15:18. doi: 10.1186/s12886-015-0008-0, PMID: 25884411 PMC4352241

[24] Ullah E McGaughey D Turriff A Sieving PA Hufnagel RB Cukras CA. (2019). Multimodal genomic analysis of hydroxychloroquine toxicity in a large cohort. Poster presentation at ARVO International Meeting, Vancouver.

[25] McConnell DG Wachtel J Havener WH. Observations on experimental chloroquine retinopathy. Arch Ophthalmol. (1964) 71:552–3. doi: 10.1001/archopht.1964.00970010568022, PMID: 14109043

[26] Rosenthal AR Kolb H Bergsma D Huxsoll D Hopkins JL. Chloroquine retinopathy in the rhesus monkey. Invest Ophthalmol Vis Sci. (1978) 17:1158–75. PMID: 102610

[27] Abokyi S Shan SW Lam CH Catral KP Pan F Chan HH. Targeting lysosomes to reverse hydroquinone-induced autophagy defects and oxidative damage in human retinal pigment epithelial cells. Int J Mol Sci. (2021) 22:9042. doi: 10.3390/ijms22169042, PMID: 34445748 PMC8396439

[28] Schmidt-Erfurth U Sadeghipour A Gerendas BS Waldstein SM Bogunović H. Artificial intelligence in retina. Prog Retin Eye Res. (2018) 67:1–29. doi: 10.1016/j.preteyeres.2018.07.004 30076935

[29] Waldstein SM Seeböck P Donner R Sadeghipour A Bogunović H Osborne A. Unbiased identification of novel subclinical imaging biomarkers using unsupervised deep learning. Sci Rep. (2020) 10:12954. doi: 10.1038/s41598-020-69814-1, PMID: 32737379 PMC7395081

[30] Dai L Wu L Li H Cai C Wu Q Kong H. A deep learning system for detecting diabetic retinopathy across the disease spectrum. Nat Commun. (2021) 12:3242. doi: 10.1038/s41467-021-23458-5, PMID: 34050158 PMC8163820

[31] Kalra G Talcott KE Kaiser S Ugwuegbu O Hu M Srivastava SK. Machine learning-based automated detection of hydroxychloroquine toxicity and prediction of future toxicity using higher-order OCT biomarkers. Ophthalmol Retina. (2022) 6:1241–52. doi: 10.1016/j.oret.2022.05.031, PMID: 35691579 PMC9722508

[32] Kim KE Ahn SJ Woo SJ Park KH Lee BR Lee YK. Use of optical coherence tomography retinal thickness deviation map for hydroxychloroquine retinopathy screening. Ophthalmology. (2021) 128:110–9. doi: 10.1016/j.ophtha.2020.06.021, PMID: 32553941

[33] Ting DSW Cheung CY Lim G Tan GSW Quang ND Gan A. Development and validation of a deep learning system for diabetic retinopathy and related eye diseases using retinal images from multiethnic populations with diabetes. JAMA. (2017) 318:2211–23. doi: 10.1001/jama.2017.18152, PMID: 29234807 PMC5820739

